# A New Method for Distinguishing Colony Social Forms of the Fire Ant, *Solenopsis invicta*

**DOI:** 10.1673/031.010.7301

**Published:** 2010-06-22

**Authors:** DeWayne Shoemaker, Marina S. Ascunce

**Affiliations:** USDA-ARS Center for Medical, Agricultural, and Veterinary Entomology, 1600/1700 SW 23rd Drive, Gainesville, Florida, 32608, United States of America

**Keywords:** assays, budding, introduced, monogynous, native, polygynous

## Abstract

Two distinct forms of colony social organization occur in the fire ant *Solenopsis invicta* Buren (Hymenoptera: Formicidae): colonies of the monogyne social form are headed by a single egg-laying queen, whereas those of the polygyne social form contain multiple egg-laying queens. This major difference in social organization is associated with genetic variation at a single gene (*Gp-9*) whereby all polygyne queens possess at least one *b*-like allele, while monogyne queens lack such *b*-like alleles and instead harbor *B*-like alleles only. Further, a recent study of native populations revealed that all *b*-like alleles in polygyne queens consistently contain three diagnostic amino acid residues: possession of only one or two of these critical residues is not sufficient for polygyny. TaqMan® allelic discrimination assays were developed to survey the variable nucleotide sites associated with these three critical amino acid residues. The assays were validated by surveying nests of known social form from the species' introduced in the USA and from native South American ranges, as well as by comparing the results to *Gp-9* sequence data from a subset of samples. The results demonstrate these new molecular assays consistently and accurately identify the variable nucleotides at all three sites characteristic of the *B*-like and *b*-like *Gp-9* allele classes, allowing for accurate determination of colony social form.

## Introduction

A major distinction in the social organization of ant societies involves the number of queens in a colony (Hölldobler and Wilson 1977): monogyne (M) colonies have only a single queen, whereas polygyne (P) colonies have multiple queens. Contrasting reproductive and dispersal syndromes often are associated with these different social systems (Ross and Keller 1995; Keller 1991; Keller and Passera 1989). For instance, M colonies ordinarily are founded by new queens independently, without workers present, whereas P colonies generally are founded through the process of budding, during which some queens and workers split off from the parent nest to establish a new nest. Newly reared queens from P colonies typically seek out established nests where, if accepted by the workers, they initiate reproduction.

Both M and P colonies occur in populations from both the native and introduced ranges of the invasive fire ant *Solenopsis invicta* Buren (Hymenoptera: Formicidae) (see Tschinkel 2006 and references therein). Remarkably, this polymorphism in social organization in *S. invicta* is associated with variation at a single gene, *Gp-9* (Ross 1997). Two classes of variants, designated as *B*-like and *b*-like alleles, occur at *Gp-9* in *S. invicta*. All P queens possess at least one *b*-like allele whereas M queens lack such *b*-like alleles and instead harbor *B*-like alleles only (Gotzek et al. 2007; Krieger and Ross 2005; Krieger and Ross 2002; Ross and Keller 1998; Ross 1997).

**Table 1.**
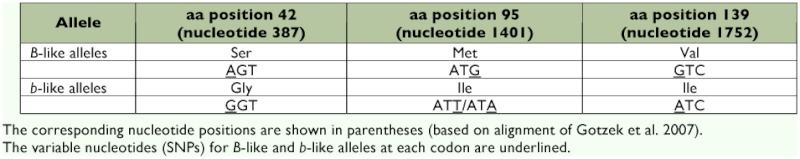
Codons of the three amino acid residues 42, 95, and 139 at *Gp-9* informative for distinguishing colony social forms of *S. invicta*.

Thus, the presence of *b*-like alleles among colony members appears both necessary and sufficient for the expression of polygyny (Gotzek et al. 2007; Krieger and Ross 2002; Ross and Keller 2002; Ross and Keller 1998; Ross 1997).

A recent molecular study by Gotzek et al. (2007) showed that a substantial amount of variation exists at *Gp-9*, indicating the presence of many *B*-like and *b*-like alleles. All described *b*-like alleles that are invariably associated with polygyny bear three diagnostic amino acid residues at positions 42, 95, and 139 (Table 1). Importantly, the discovery of several unique alleles with various combinations of *b*-like and *B*-like codons, coupled with data regarding the social organization of the source colonies for these alleles, revealed that no single *b*-like residue at positions 42, 95, and 139 is completely predictive of P behavior: *b*-like residues invariably were present at all three positions in sequences from all P colonies, suggesting that all three *b*-like amino acids are necessary for the expression of polygyny (Gotzek et al. 2007).

The two social forms of *S. invicta* differ not only in colony queen number and *Gp-9* genotype but also in important features of their reproductive and dispersal behaviors. In the M form, sexuals take part in aerial mating flights by ascending to elevations of 100 m or more for pairing and may be transported several kilometers or more by wind currents during these flights (Markin et al. 1971).

Mating swarms in the P form occur at lower elevations (often at head-height), and the vagility of queens of this form seems correspondingly more restricted than in the M form (Goodisman et al. 2000; DeHeer et al. 1999). An important additional means of natural spread of the P form is through colony budding or fissioning, a process in which workers and queens from a parent colony travel on foot to establish a new colony (Vargo and Porter 1989). These differences in reproductive and dispersal behaviors are expected to have a number of important effects on the distribution of genetic variation at various spatial scales, as well as management strategies employed for population suppression or local eradication. Thus, the ability to accurately classify colonies to social form is critical to management strategies, to constructing predictive models of their spread and expansion, and to assessing the potential of successful eradication of *S. invicta* in newly invaded areas (Drees and Vinson 1990).

Several assays have been developed and used previously to score *Gp-9* genotypes of fire ants. All of these methods have shortcomings, the most significant of which is that none of these previous methods jointly survey variation at all three diagnostic amino acid residues. The original discovery and early studies of *Gp-9* involved scoring genotypes of individuals from the protein product of *Gp-9* detected by protein electrophoresis using a non-specific protein stain (DeHeer et al. 1999; Ross 1997). However, later studies revealed this method cannot distinguish all *b*-like alleles because a subset have the same electrophoretic mobility as many *B*-like alleles. An additional limitation of this method is that the protein product of *Gp-9* is not detectable in brood or in males of any developmental stage and only becomes detectable in adult females at a minimum posteclosion age of 8–14 days. Ross et al (2003) designed two different two-stage PCR assays that distinguish *b*-like from *B*-like sequences in all fire ant species. However, in addition to being time consuming, these assays jointly survey variation at codons 95 and 139 only, without surveying variation at codon 42. The same holds true for the recently developed competitive allele-specific PCR (Imyanitov et al. 2002) method developed by Gotzek et al (2007). Finally, Valles and Porter (2003) developed a simple molecular assay to differentiate social forms in *S. invicta* involving multiplex PCR using allele-specific primers for *B*- and *b*-alleles. Using this assay, PCR amplification of DNA from *BB* (M or P) ants results in a single 517 bp amplicon (specific for *B* alleles), whereas PCR amplification of DNA from *Bb* ants results in both 517 bp and 423 bp amplicons (specific for *B* and *b* alleles, respectively). While this multiplex PCR assay is widely used and appears to be reliable in distinguishing social forms in US populations of *S. invicta* where few *Gp-9* allelic variants exist (as are the other methods above), this assay cannot distinguish social forms reliably in the native South American range, where many *Gp-9* variants exist, simply because it does not provide information on variation at all three diagnostic amino acid residues. Specifically, allele-specific PCR using the *B*-specific primers 26BS and 16BAS does not survey variation at any of the three diagnostic amino acid residues (neither the primers nor amplicons include sites corresponding to these residues) but instead corresponds to variation at another amino acid residue (position 152) that is only partially diagnostic in distinguishing the two social forms (Gotzek et al. 2007). Further, allele-specific PCR using the *b*-specific primers 24bS and 25bAS effectively surveys only one of these three diagnostic amino acid residues (the nucleotide on the 3'end of primer 24bS corresponds to the variable nucleotide site at amino acid residue 95).

The goal of this study was to develop a new molecular assay to reliably distinguish the M and P social forms of *S. invicta* and related species that effectively and efficiently surveys all three diagnostic amino acid residues invariably associated with polygyny. To accomplish this goal, the 5′-nuclease allelic discrimination assay, or TaqMan® assay, which is a PCR-based assay that has been shown to be useful for genotyping single nucleotide polymorphisms (SNPs; Livak 1999), was utilized. For this assay, the region flanking a SNP is PCR-amplified in the presence of two allele-specific fluorescent probes. Each probe is specific to one of the two alleles associated with a particular SNP. Discrimination of alleles using the TaqMan® assay eliminates the need for post-amplification steps and is less technically demanding and more rapid than conventional PCR methods (Livak 1999). The new method is robust, sufficiently high-throughput, and accurate for determining social form of fire ant colonies.

## Materials and Methods

### DNA samples

Total genomic DNA was extracted from either single individuals or groups of individuals (bulk extractions) using the Puregene DNA isolation kit (Gentra Systems, www.gentra.com). The 99 bulk extractions of ants each consisted of 10–15 ants from a given nest, and the 75 new extractions were from single individual workers. Source material was derived from colonies of both social forms collected in both the introduced and native geographic ranges.

### Design of primers and probes for TaqMan® allelic discrimination assays

Regions for designing primers and probes within the *Gp-9* gene were selected using the sequence alignment from Gotzek et al. (2007). This alignment includes a total of 185 sequences, 136 of which were obtained from *S. invicta* collected over much of its native range, as well as sequences from several other *Solenopsis* species. Conserved regions around the three variable nucleotide positions corresponding to the amino acid changes at positions 42, 95, and 139 (see Table 1) were identified, and primer pairs within flanking conserved regions, as well as two probes for each single nucleotide polymorphic site (SNP), were designed using Primer Express version 3 software (default parameters, Applied Biosystems, www.appliedbiosystems.com). To increase specificity, each designed probe was labeled with a reporter dye (either FAM or VIC), conjugated to a minor groove binder group (Kutyavin et al. 2000) and a quencher at the 3′ end, that made it specific to one of the two possible nucleotides at each informative position (primers purchased directly from Applied Biosystems). A third probe was designed for the assay corresponding to the variable nucleotide site for the residue at position 95 because of the presence of a third nucleotide variant at this position (Table 1). Sequences of primers and probes are shown in Table 2.

### TaqMan® allelic discrimination assays

**Figure 1.**
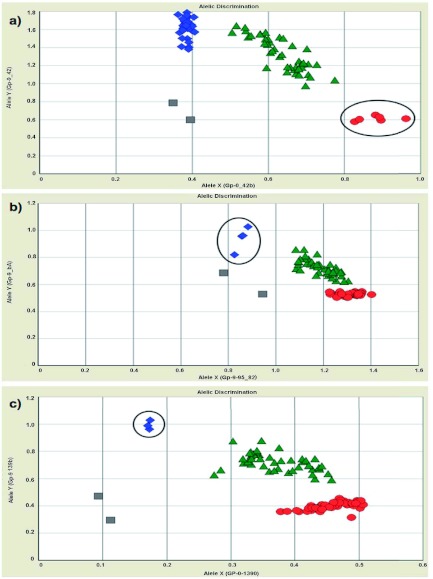
Allelic discrimination X–Y bivariate plots for the three TaqMan® assays. Each colored symbol in each panel represents a single *Solenopsis invicta* sample. The position of a given symbol is defined by the fluorescence reading obtained for the two fluorogenic probes: The X-axis represents the relative fluorescent emission for the allele-specific probe labeled with VIC, and the Y-axis represents the emission for the allele-specific probe labeled with 6-FAM. a) TaqMan® assay for nucleotide position 387 (corresponding to amino acid position 42). b) TaqMan® assay for nucleotide position 1401 (amino acid position 95), c) TaqMan® assay for nucleotide position 1752 (amino acid position 139). Red dots, homozygous *BB*; blue diamonds, homozygous *bb*; green triangles, heterozygous *Bb*. Grey squares represent no template controls. Individuals of *bb* (or *b* in case of haploid males) genotype are enclosed in black circles. High quality figures are available online.

After performing several optimizations, the final assay conditions were as follows: 2.5 µl of 1X TaqMan® Genotyping PCR Master Mix (Applied Biosystems), 0.2 µM sense and anti-sense primers, 0.05–0.2 µM of each probe (Applied Biosystems) (see Table 2 for concentrations of each probe), 1 µl of genomic DNA (≈ 20 to 200 ng/µl) or water for a no-template control, and PCR grade water to bring final volume to 25 µl. Reactions were set up in a MicroAmp® 96-well optical plate (Applied Biosystems) and sealed with MicroAmp® optical film (Applied Biosystems). All PCR amplifications were carried out under the following thermal cycling conditions: 10 min at 95° C, followed by 40 cycles at 95° C for 15 s and 60° C for 60 s in either the ABI Prism 7500 Sequence Detection System or Veriti Applied Biosystems thermal cycler. For all experiments, positive controls for each probe (homozygous *BB*; homozygous *bb* and heterozygous *Bb*) and 1–3 negative controls (water) were included on each 96-well plate alongside the study samples. Results were analyzed on the ABI Prism 7500 Sequence Detection System machine with the Real-Time PCR System Sequence Detection Software v1.3.1 (Applied Biosystems), using the allele discrimination plate read function to detect the end-point fluorescence in each well. Results were expressed as fluorescence intensity and were displayed on an X-Y bivariate plot (see Figure 1). Clusters of genotypes were manually assigned after the reporter molecule for each allele was defined.

**Table 2.**
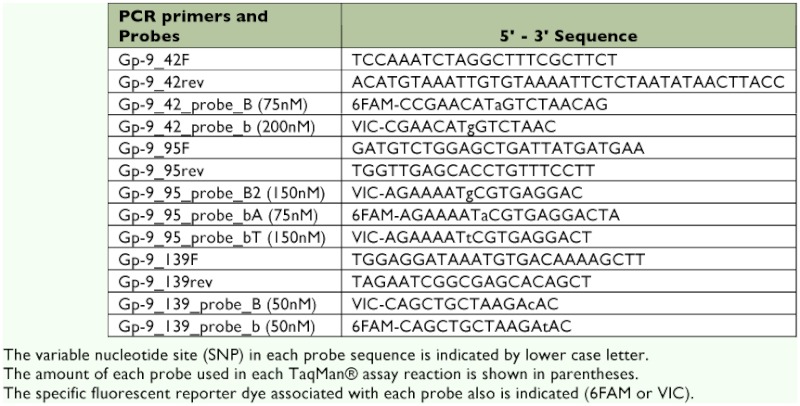
Sequences of newly designed primers and probes for each TaqMan® assay.

### Multiplex PCR using Valles and Porter (2003) assay

The multiplex PCR method of Valles and Porter (2003) was used to genotype every individual or bulk extracted DNA sample used for the TaqMan® assays above to allow for comparing the results from the two methods. PCR reactions contained 2X Hot-Start Taq Mastermix (Denville Scientific, www.denvillescientific.com), a variable amount of each primer (26BS and 16BAS; 24bS and 25bAS), 1–2 µl of total genomic DNA (≈ 30 to 200 ng/µl), and water to a final volume of 15 µl (see Valles and Porter 2003). Multiplex PCR was performed using the following parameters: 2 min at 94° C, followed by 35 cycles at 94° C for 15 sec, 55° C for 15 sec, and 68° C for 30 sec. The resulting PCR products (5 µl) were separated on 1.5–2% agarose gels and visualized by ethidium bromide staining. For all experiments, *BB* and *Bb* positive controls and a negative control (water) were PCR-amplified and run alongside the study samples.

## Results

Primers and probes were successfully designed for all three SNPs using the default parameters of the Primer Express version 3 software (Applied Biosystems). Optimal PCR reaction conditions and thermal cycling profiles were the same for all three assays, but the optimal probe concentrations did vary across assays (see methods and Table 2). An initial survey of DNA from a small set of samples of known *Gp-9* genotypes (validated by direct sequencing; see Gotzek et al. 2007) demonstrated that each assay was robust and specific in detecting the underlying SNP at each site. Potential cross-hybridization of the allele-specific probes was tested by setting up TaqMan® assay reactions containing only one of the two probes and then surveying DNA from individuals of known genotypes. The results show that cross-hybridization of probes appeared to be negligible as evidenced from the complete lack of detection of fluorescence of each probe on alternate genotypes (i.e., Primer pair Gp-9_42F/ Gp-9_42rev and probe Gp-9_42_probe_b amplified and produced increased fluorescence only for known *bb* samples but not *BB* samples and vice versa; the same held true for the other two assays).

After optimization and initial validation, the three TaqMan® allelic discrimination assays and the multiplex PCR method of Valles and Porter (2003) were used to genotype 174 *S. invicta* samples collected from the introduced and native ranges. A subset of these samples included individuals with unique alleles bearing various combinations of *b*-like and *B-*like codons at the three informative amino acid residues (19 individuals from ten colonies; see Table 3). The TaqMan® assay corresponding to amino acid residue at position 42 failed to amplify the *b* allele in only four cases (∼2% failure; all amplified successfully on second attempt). The two TaqMan® assays corresponding to residues 95 and 139 successfully detected known alleles in all cases (100% accuracy). Use of the multiplex PCR protocol of Valles and Porter (2003) for genotyping these same samples resulted in six PCR reactions that failed to produce visible products (∼3% failure). Importantly, the TaqMan® allelic discrimination assays for 19 of these DNA samples previously sequenced for the entire *Gp-9* gene, 13 of which had various combinations of *b*-like and *B*-like codons at the three informative amino acid residues, were 100%) concordant in identifying the appropriate nucleotides at each of the three positions surveyed (Table 3). In contrast, surveys of these same samples using the Valles and Porter (2003) assay revealed one case where an individual from an M nest (with two of the three nucleotides characteristic of *b*-like alleles) was misclassified as originating from a P nest based on the fact that both the 517 bp and the 423 bp amplicons were present. Additionally, this assay did not detect the rare third allele known to occur at position 95 (see Table 3). However in these cases, designation to social form was still correct.

## Discussion

Colony social organization in the fire ant *S. invicta* is associated with variation at a single gene, *Gp-9* (Ross 1997). Two classes of variants, designated as *B*-like and *b*-like alleles, occur at *Gp-9* in *S. invicta*. All individuals from M colonies possess *B*-like alleles only. In contrast, P colonies invariably contain some proportion of individuals (and all reproductive queens) possessing a *b*-like allele as well as a *B*-like allele (Gotzek et al. 2007; Krieger and Ross 2005; Krieger and Ross 2002; Ross and Keller 1998; Ross 1997). Importantly, all described *b*-like alleles that are invariably associated with polygyny bear three diagnostic amino acid residues at positions 42, 95, and 139 (Gotzek et al. 2007). Thus, no single *b*-like residue at any of these positions is completely predictive of polygyne behavior (Gotzek et al. 2007).

The above results indicate that any molecular methods developed to accurately genotype fire ants and categorize nests to social form based on *Gp-9* variation must provide information on the underlying nucleotide variation at all three informative sites corresponding to these three diagnostic amino acid residues. Said another way, the discovery of unique *Gp-9* alleles with various combinations of *b*-like and *B*-like codons, many of which are associated with monogyny (see Table 3), means that surveying variation at only one or two of these sites may lead to erroneous classification of nests to colony social form. For this study, TaqMan® allelic discrimination assays were developed to reliably distinguish the M and P social forms by effectively and efficiently surveying underlying nucleotide variation corresponding to all three diagnostic amino acid residues associated with polygyny. The assays yield highly reproducible results and are consistently accurate. Indeed, these assays were 100%) accurate in identifying the nucleotides residing at all three sites characteristic of the *B*-like and *b*-like *Gp-9* alleles of all surveyed samples, including individuals bearing various combinations of *b-*like and *B*-like codons at the three informative amino acid residues for *Gp-9*. The previously developed multiplex PCR method of Valles and Porter (2003) may be inaccurate for this purpose simply because the method effectively surveys only one of the three informative sites at *Gp-9* (corresponding to amino acid residue at position 95). This is evident from the survey of nests composed of individuals bearing combinations of *b*-like and *B*-like codons at the three informative amino acid residues in which a single nest was incorrectly classified as polygyne using this method (Table 3). Additionally, this method does not detect the rare third allele known to occur at position 95 (see Table 3). While a strong case still could be made that the Valles and Porter (2003) method is robust for determining social form of nests in the USA, where few alleles at *Gp-9* exist (and no evidence for mosaic alleles), clearly the method is not reliable for surveys of nests from the species' native range or for surveys conducted in other introduced areas (e.g., Taiwan, China, Australia) where the source population is unknown. Thus, results of studies in these areas employing this method for determining colony social form should be interpreted with caution.

**Table 3.**
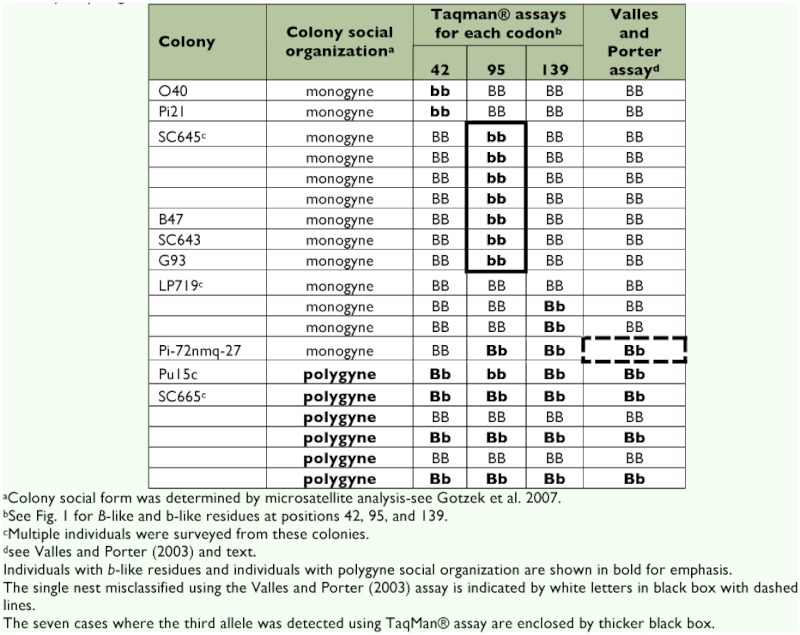
Results for TaqMan® allelic discrimination assays for SNPs corresponding to amino acid residues at three *Gp-9* codons jointly diagnostic for *B*-like and *b*-like alleles.

While there is no replacement for surveying nucleotide variation at all three informative sites, one could use a two-fold approach for surveying nests of unknown social form whereby genotype at one of the three variable sites is first determined using one of the TaqMan® allelic discrimination assays, followed by subsequent screening of individuals that bear at least one *b*-like residue using the two additional assays corresponding to the other SNPs. For example, if one detects a nucleotide characteristic of a *b*-like allele (associated with polygyny) on first pass with a single assay, then subsequent employment of the other two allelic discrimination assays could be performed to confirm that nucleotides corresponding to *b*-like residues were also present at these positions as well, confirming that the sample is from a polygyne nest. On the other hand, if an initial survey of an unknown sample (preferably DNA from a bulk sample of 10 ants since not every worker bears a *b*-like allele in a polygyne nest; see Table 3) reveals the presence of *B*-like alleles only, then further screening is unnecessary since the colony is presumably monogyne. While this approach potentially may save time and resources, setting up all three assays simultaneously is preferable since information at all three sites is obtained, providing an internal control in most cases, and this requires only minimal extra effort.

In summary, the TaqMan® allelic discrimination assays were 100% accurate in identifying the appropriate nucleotides at each of the three informative nucleotide positions surveyed at *Gp-9* necessary for the expression of polygyny in *S. invicta*, allowing for rapid and accurate classification of nests as belonging to either the M or P social form. In addition to being robust and accurate, these assays also have several other advantages over previous methods, including shorter reaction preparation time (less than three hours for 96 samples), less post-amplification manipulations (reducing the potential of PCR product contamination), the lack of need for special handling of ethidium bromide, greater sensitivity and reproducibility, and automated scoring of genotypes, which subsequently can be exported as an editable text file.

## Abbreviations

P: polygynous;
M: monogynous;
SNP: single nucleotide polymorphism site
